# Chemo-treated 4T1 breast cancer cells radiation response measured by single and multiple cell ionization using infrared laser trap

**DOI:** 10.1038/s41598-019-53821-y

**Published:** 2019-11-26

**Authors:** Endris Muhammed, Li Chen, Ying Gao, Daniel Erenso

**Affiliations:** 1 Department of Physics, Addis Ababa University, Addis Ababa, 1176 Addis Ababa, Ethiopia; 2 Department of Pharmacology, College of Medicine, Guangxi University of Science and Technology, Liuzhou, Guangxi 545006 China; 3 International Ginseng Institute, School of Agriculture, Middle Tennessee State University, Murfreesboro, TN 37132 USA; 4 Department of Physics, Middle Tennessee State University, Murfreesboro, TN 37132 USA

**Keywords:** Breast cancer, Optical manipulation and tweezers, Radiotherapy, Chemotherapy

## Abstract

We present a study that uses a laser trapping technique for measurement of radiation sensitivity of untreated and chemo-treated cancer cells. We used a human mammary tumor cell line (4T1) treated by an antitumor compound, 2-Dodecyl-6-methoxycyclohexa-2, 5-diene-1,4-dione (DMDD), which was extracted from the root of Averrhoa carambola L. The untreated control group, and both 2-hour and 24-hour treated groups of 4T1 cells were used in this study. The absorbed threshold ionization energy (TIE) and the threshold radiation dose (TRD) were determined using a high-power infrared laser (at 1064 nm) trap by single and multiple cells trapping and ionization. The results were analyzed using descriptive and t-statistics. The relation of the TIE and TRD to the mass of the individual cells were also analyzed for different hours of treatment in comparison with the control group. Both TIE and TRD decrease with increasing treatment periods. However, the TRD decreases with mass regardless of the treatment. Analyses of the TRD for single vs multiple cells ionizations within each group have also consistently showed this same behavior regardless of the treatment. The underlying factors for these observed relations are explained in terms of radiation, hyperthermia, and chemo effects.

## Introduction

According to the International Agency for Research on Cancer (IARC) 2018 report, there are nearly 2.1 million new cases and 0.5 million deaths of breast cancer worldwide^1,2^ annually. Although breast cancer ranks second, behind lung cancer, in the number of new cancer cases (12%), it has a relatively low death rate (7%), ranking fifth next to liver cancer. This higher survival rate might be credited to the advancement of traditional therapies such as radiotherapy^3,4^ chemotherapy^5–8^, surgery^9^ and to the development of relatively newer therapies such as hypothermia and hyperthermia that uses nanotechnology^10–13^ and immunotherapy^14^.

Radiotherapy (RT) is one of the effective tools used for different malignant tumors. It is used to kill all the cancer cells in a tumor with a radiation energy that is enough to overcome the electron binding energy in an atom or a molecule. In RT when such radiation energy to treat a patient it can cause unintended damage to the normal cells surrounding the tumor. Therefore, the goal of RT must then be to maximize the radiation damage to the cancer cells with no or minimized compromise to the integrity of the normal cells. It is well known that a combined modality treatment, such as radiation and chemo therapies is better than RT alone as such treatment enhances death of tumor cells through an inhibition of DNA repair processes^8^. Some chemotherapeutic drugs can make tumor cell clonogens to be more susceptible to ionization energy so that a reduced radiation dose is needed to kill the tumor cells. Cisplatinum and taxanes are the standard chemotherapeutic agents that have been used for such improved therapeutic outcomes in radiation and chemo combined modalities. However, using these agents in radiation and chemo combined modalities limits the radiation dose as these agents are commonly associated with considerable toxicity to normal tissues^8^. Other recently developed combined modalities of treatment involve hypothermia, hyperthermia, and biocompatible nanoparticles. Studies in 4T1 breast cancer cells in mice have shown hypothermia and hypoperfusion effects induced by pacilitaxel (PTX) that maintain reduced body temperature may prevent tumor relapse or metastasis after chemotherapy^11^. It has also been shown that biocompatible nanoparticles, such as gold silica nanoshells (GSNs) designed to absorb light in the infrared which has a high tissue transparency, have been used to treat prostate cancer^13^. Particles such as GSNs have the ability to absorb the infrared light and to generate heat which induces highly localized hyperthermia, a modality shown to be highly effective for photothermal cancer therapy. Thus, strategies for combined modalities of cancer treatment that utilize radiotherapy, chemo, and possibly hyperthermia effects could provide a new approach for better efficacy of such treatments. Some studies have shown that traditional Chinese medicines (TCMs) used to treat various types of cancer contain antitumor agents that could increase the radiation sensitivity of tumor cells and provide protection against radiation-induced damage in normal tissues^15,16^.

In this paper we are presenting a study we conducted on the radio-sensitivity of 4T1 breast cancer cell lines treated by an antitumor herbal extract from TCM under hyperthermia conditions. This study uses a new approach that uses laser trapping technique (LT)^17^ for single cell ionization. We have recently demonstrated this approach in BT20 breast cancer cells^18^ and human red blood cells^19,20^. This study uses a high-power infrared laser (at 1064 nm) to measure the radio sensitivity of 4T1 breast carcinoma cell lines treated by a naturally occurring compound, 2-Dodecyl-6-methoxycyclohexa-2, 5-diene-1, 4-dione (DMDD) extracted from the root of *Averrhoa carambola* L. Studies have shown that DMDD induces apoptosis of various human breast cancer cells through production of intracellular reactive oxygen species (ROS) and inhibition of NF-κB activation^21^. Recent studies, using an *in vivo* mouse model of transplanted 4T1 breast cancer cells, have also shown that DMDD effectively suppressed the growth of primary breast tumor and simultaneously inhibited the metastasis of breast tumor to the lung and liver, as well as prolonged the survival of tumor-bearing mice^22^. The 4T1 breast carcinoma cell line is highly tumorigenic and invasive; it can metastasize from the primary tumor to multiple distant sites such as lymph nodes, liver, lung, brain, and bone^22^. As the progressive metastases of 4T1 to the lymph nodes and other sites are very similar to the clinical situation of human breast cancer, it makes 4T1 an ideal experimental model for human metastatic breast cancer. Our study investigated a statistically significant number of untreated 4T1 breast cancer cells (as a control group) and two groups treated by DMDD for two and twenty-four hours. The radio-sensitivity of each cell from these three groups was studied by calculating the threshold radiation dose for each group. Ionization by laser trap in the infrared region could lead to hyperthermia damage due to the absorption of infrared wavelength radiation by the water molecules in the cell and the surrounding suspension medium. Furthermore, field damage induced by ionizing radiation could play a significant role in the determination of the threshold radiation dose. Our study investigates both hyperthermia and induced charge effects by estimating the threshold radiation for both the control and treated groups, and by introducing a new multiple cell trapping approach that we present here for the first time.

## Methods

### Cell culture and treatment

4T1 cells were cultured in RPMI1640 medium with 10% FBS in a 5% CO_2_ and 37 °C incubator. Cells were trypsinized and passaged every 2–3 days. After 4T1 cells were trypsinized, they were diluted with RPMI1640 medium, and seeded in a 96-well plate with an intensity of 5,000–7,000 cells per well (100 μL/well). After the cells were attached to the bottom of the wells for 24 h, cells were treated with DMDD at 100 μM for 2 or 24-h. Each of the untreated group, 2-h treatment group, and 24-h treatment group had six replicate wells. Following treatment, the culture medium in each well was transferred to an Eppendorf tube. Subsequently, wells were rinsed with PBS and 50 μL trypsin was added to each well, and the detached cells were transferred to the same Eppendorf tube.

### Laser trap set-up

**Figure 1:**
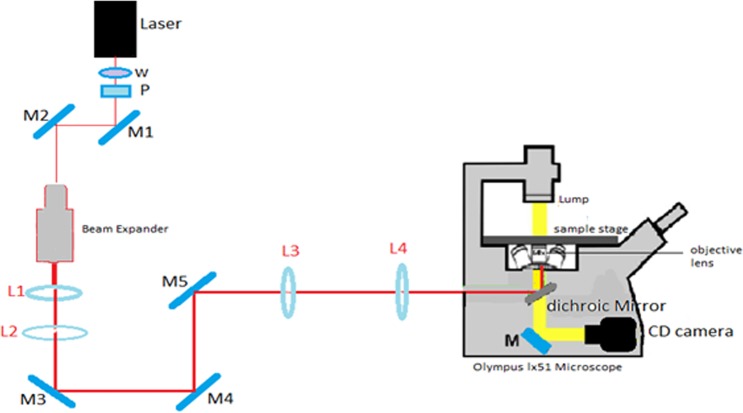
Schematic of the laser trap experimental set-up. The laser has a wavelength 1064 nm and maximum power 8 Watts. The power is controlled by a half-wave plate (W) and a polarizer (P). The mirrors M1 and M2 are used to redirect the laser beam. The beam expander, made of two converging lenses, is used to increase the beam diameter. Another two converging lenses (L1 and L2) further readjust the beam diameter so that beam diameter can fit to the diameter of the window of the objective lens. The mirrors M3 and M4 are used for alignment and redirecting of the laser beam. M5 is a steering mirror along with the converging lenses (L3 and L4) to control the location of the trap on the focal plane of the microscope.

The set-up for the laser trap is shown in Fig. 1. This experimental set-up is very similar to the set-up used in previous biomedical laser trapping application studies^18–20^. Thus, here we briefly discuss the basic elements of this set-up relevant to our study. The laser we used is infrared diode-pumped laser lasing at 1064 nm (Spectra physics V-extreme Nd:YVO4 laser). It generates a linearly polarized beam with a maximum power of 8 W and beam size of 4 mm. A polarizer (P) was used to control the power of the beam. The beam directed by the mirrors M1 and M2 passes through a 20X beam expander and a pair of lenses (L1 and L2) with 5 cm and 20 cm focal length, respectively, to increase the beam size to about the diameter of the window of the objective lens of the microscope (~2 cm); this expansion is critical for a stronger trap. Mirrors M3 and M4 were used for redirecting and better alignment of the beam while M5 was positioned at a proper distance to create a steerable trap at the focal plane of the microscope. The position of M5 was 20 cm away from the third converging lens (L3) that is positioned from another converging lens (L4) with the same focal lengths of 20 cm placed 20 cm from the back of the objective lens. L3 and L4 were separated by a distance of twice their focal length so that the conditions from geometrical optics are satisfied for the formation of a steerable trap on the focal plane of the microscope. Then, the collimated and aligned beam was coupled to the microscope via a Dichroic mirror (DM) positioned at 45 degrees inside the microscope. The DM reflects the laser beam for a normal incidence at 100X objective lens with a 1.25 numerical aperture. At the same time, the DM transmits the imaging light from Olympus Tl4 halogen lamp for the live image captured by a PC-controlled digital camera integrated to the microscope via the second port of the microscope. For experiments conducted in trapping and ionizing a cell, the power was measured at two positions. One is before the beam is incident on L4 (~4.34 W) and the second is after exiting the objective lens (~0.806 W). An efficiency of about ~18.57% was maintained throughout our measurements.

### The mechanism of single cell ionization

The cell membrane controls the movement of the substances between the intracellular region and the environment of the cell. The main component of the cell membrane is phospholipids. The lipids contain polar phosphate molecules (hydrophilic) and non-polar molecules (hydrophobic) composed of a relatively straight alkyl chain group ranging from 17–25 carbon atoms long^23^. Lipids in aqueous solution spontaneously form a sheet-like bilayer structure two molecules thick, with the polar head portion outside and the non-polar alkyl chain pointing inside. The contacting polar head with water dissolves the ions (Na^+^, K^+^, and Cl^-^) surrounding the bilayer. The concentration gradient of ions at opposite ends of the membrane forms an electrostatic potential difference across the cell. When an external field is applied it causes an extreme polarization that leads to a torque due to the misalignment of the induced dipoles. The membrane structure is rearranged to form aqueous pores which increase the conductivity and permeability of the membrane so that the membrane enables water and molecular transport to the cell, which is known as reversible electroporation^24,25^. However, if the exposure is to a strong and rapidly oscillating electric field (such as that generated by the laser field), the membrane does not reseal and electroporation becomes irreversible. The electrons are pulled apart from the atoms completely and permanently, resulting in the ionization of the cell. The 4T1 cells’ membrane, first damaged by the drug to some extent, is exposed to such a field when it is trapped by the laser. While the cell is in the trap a gradual buildup of charge on the cell takes place that results in a gradually increasing electrostatic force. At some instant of time, when the cell undergoes complete dielectric breakdown, the strength of this force overcomes the gradient trapping force and the cell is ejected from the trap. During this period of time, the amount of the radiation energy of the laser incident on and absorbed by the cell determines the threshold ionization energy needed to kill the cell.

### Threshold ionization energy and radiation dose

**Figure 2:**
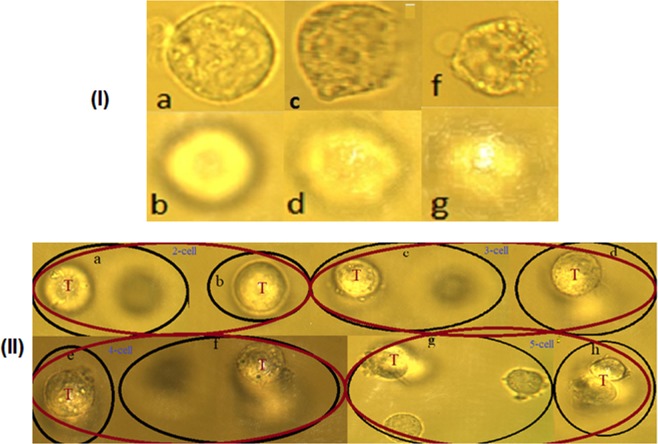
(I) *Single cell ionization*: The images captured for 4T1 breast cancer cells before and after trapping: (**a**,**b**) for the untreated control; (**c,d**) for 2-h treated; (**f,g**) for 24-h treated, respectively. (II) *Multiple cell ionization*: for the untreated 2-cells, 3-cells, 4-cells, and 5-cells ionization, the letter (**T**) represented the trapping point. For 2-cells (**a**) one trapped the second is free, (**b**) both trapped. For 3-cells (**c**) two trapped and one free, (**d**) all three cells trapped. For 4-cells (**f**) three trapped and one free, (**e**) all four cells trapped. For 5-cells (**g**) three trapped and two free, (**h**) all five cells trapped.

The 4T1 cells from the untreated control, the 2-h and 24-h treated groups were prepared in a well slide and mounted on the microscope micro-driven mechanical stage. Using the digital camera, we took an image of the cell when free and lying on the bottom of the slide. We then opened the gate at the laser port of the microscope and trapped the cell. Successive images of the cell were taken by the digital camera at a fixed frame grabbing rate until the cell is fully ionized, ejected from the trap, and disappeared from the camera view range. Real live successive images illustrating this process are shown in Fig. 2a,b for untreated, Fig. 2c,d for 2-h treated, and Fig. 2f,g for 24-h treated. Our objectives were to study the radiation sensitivity of the 2-h and 24-h treated in comparison with the untreated control group by calculating and analyzing the threshold radiation dose (TRD) for the 4T1 cells. The TRD depends on the corresponding mass and the average radiation energy absorbed by each cell.

Spherical models for 4T1 cells in all the three groups were considered and the diameter of each cell was determined for each free cell using the software ImageProplus6 in pixels. A conversion factor of 7.27 × 10^−8^ *meter/pixel*, was found using 3.1 μm silicon beads. This conversion factor was used to determine the cross-sectional area, *A*_*cell*_, and the volume for each cell, *V*_*cell*_. The mass of each cell,

1 $${M}_{cell}=\rho {V}_{cell}$$

was calculated by using the widely accepted density of cancer cells, *ρ* = 1000 *kg*/*m*^3 ^^26–28^. TRD was determined using the mass calculated for each cell and the average radiation energy absorbed. The average power incident on the cell, which was kept the same throughout the ionization of each cell, was P_I_ = 0.806 W and the average estimated transmitted power was, P_T_ = 0.64 W. We then determined the average threshold ionization energy for each cell,

2 $$TIE=\frac{{A}_{cell}}{{A}_{beam}}\,({P}_{I}-{P}_{T})\,T,$$

where *T* is the ionization period that was determined using the digital camera image grabbing rate and the number of images captured during the time covering the instant each cell entered and the instant it got ejected from the trap. ${A}_{{beam}}$ is the beam size determined at the trap location using the numerical aperture of the objective lens^29^. The TRD was then calculated for each of the 4T1 cells using

3 $$TRD=TIE/{M}_{cell}$$

here the TIE is the minimum radiation energy absorbed by the cell that creates a significant amount of charge through ionization that leads to irreversible damage to the cell. When the cell reaches this threshold charge, it experiences a stronger electrostatic force than the trapping force that results in the ejection of the cell from the trap^18–20^.

## Results and Discussion

### Single cell ionization

A total of 89, 86, and 128 4T1 cells for the control untreated, 2 h, and 24 h treated groups were studied, respectively. For each of these groups, the basic statistical parameters for the average ionization time ($\overline{T}$), cross-sectional area $({\overline{A}}_{cell})$, volume $({\overline{V}}_{cell})$, mass $({\overline{M}}_{cell})$, TIE, and TRD are given in Table 1. The values for the average ionization time calculated in Table 1 clearly show that as the treatment period increases, the ionization time decreases. On the other hand, the average cross-sectional area and the volume calculated using a spherical model do not display significant differences. This also results in similar values for the calculated average masses in the three groups. These close values indicate that any variations in response to radiation for the three groups are primarily affected by inherent biochemical structure differences caused by the dose and period of treatment of DMDD.

**Table 1:** Basic statistical parameters for the measured and calculated quantities: Area, Mass, TIE, and TRD.

Quantities	Mean	SD	Min.	Median	Max.
**Untreated 4T1 control group (89 Cells)**					
T (sec)	341.2	145.8	180.0	286.8	780.0
A_cell_ (μm)^2^	204.2	72.1	55.0	197.1	475.6
M_cell_ (ng)	2.3	1.3	0.3	2.1	7.8
V_cell_ (µm)^3^	2291.6	1290.5	306.9	2082.7	7804.6
TIE (mJ)	81.3	63.5	24.6	64.6	440.1
TRD (J/μg)	32.6	14.8	15.8	27.0	88.6
**2-h treated 4T1 group (86 Cells)**					
T (sec)	225.4	112.3	96.0	188.4	579.0
A_cell_ (μm)^2^	193.6	76.0	60.0	192.0	397.0
M_cell_ (ng)	2.1	1.2	0.6	2.0	6.0
V_cell_ (µm)^3^	2142.1	1239.5	349.7	2002.8	5952.0
TIE (mJ)	48.8	37.7	10.5	32.2	192.2
TRD (J/μg)	22.4	11.4	9.4	18.6	55.6
**24-h treated 4T1 group (128 Cells)**					
T (sec)	102.5	70.0	31.2	76.8	406.8
A_cell_ (μm)^2^	209.8	68.5	68.0	200.5	372.0
M_cell_ (ng)	2.4	1.1	0.4	2.1	5.4
V_cell_ (µm)^3^	2377.0	1144.1	421.9	2136.2	5398.7
TIE (mJ)	23.8	20.4	4.0	15.2	115.5
TRD (J/μg)	9.4	6.4	2.7	7.7	46.9

**Figure 3:**
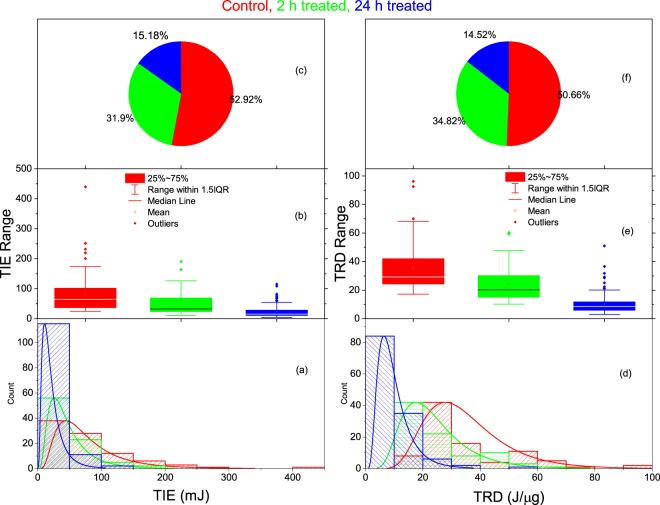
The statistical distributions for the TIE (**a–c**) and the TRD (**d,e**) for untreated control group (red), 2-h treated group (green), and 24-h treated group (blue) 4T1cells.

Using the ionization time, each cell area, the beam at the trap location, the power incident, and the power transmitted, we have calculated the absorbed threshold ionization energy using Eq. (2). The average values, for the untreated control, 2-h, and 24-h treated groups, the average absorbed threshold ionization energy, were found to be *TIE* = 81 ± 64.00 *mJ* 49 ± 38 *mJ* and 24 ± 20 *mJ* respectively. The higher standard deviation for the three groups is due to the wide range of cell size, one of the factors that affects the magnitude of the ionization energy. The statistical distributions for TIE for all cells in each group are displayed using pie charts, box plot, and histograms in Fig. 3(a–c). In each graph the data coded red represents the untreated control group, green denotes the 2-h treated group, and blue denotes the 24-h treated 4T1 cells. From these distribution graphs and the calculated average values, we can clearly see that the TIE for the treated groups is less than the untreated control group. This effect is amplified with an increase in the duration of treatment as we can see from the lower TIE for the 24-h treated group than the 2-h treated group as shown in Fig. 3(b). The pie chart in Fig. 3(c) shows the TIE for untreated 4T1cells makes up 53.15% of the pie, whereas the 2-h and 24-h treated 4T1 cells cover 31.7% and 15.15%, respectively. We have used a different number of cells in each group. The absorbed TRD for each cell in each group is calculated using the masses in Eq. (1) and the TIE in Eq. (3). The results for each group, using the same color coding, are also displayed in Fig. 3(d–f). For the untreated control, the 2-h treated, and 24-h treated groups, the average absorbed TRD were found to be *TRD* = 33 ± 15*J*/*μg* 22 ± 11*J*/*μg*, and 10 ± 6*J*/*μg*, with 53.49%, 32.38%, and 14.12% respectively depicted in the pie chart shown in Fig. 3(f). This Figure also shows lower radiation doses in the 4T1 cells treated with DMDD than the control group. This effect is also amplified with the treatment period increase as shown in Fig. 3(e).

Further validity of the results obtained for TIE and TRD is confirmed with independent two-sample t-statistical analyses using Origin2018 programing software. Generally, in t-statistics the t-test procedure automatically provides two tests of the mean difference. One is based on the assumption that the variances of the two samples are equal and the other not equal (Welch correction). These tests are often referred to as “unpaired” or “independent samples” t-tests, as they are typically applied when the statistical units underlying the two samples being compared are non-overlapping which is the case for the results obtained for both the TIE and TRD. The results for t-test statistics for the TIE and TRD are given in Table 2. These results show that there is a significant difference between the control group and the 2-h treated, the control group and the 24-h treated, and between the 2-h treated and 24-h treated groups. At 0.01 level (99% confidence), in all the three independent two-sample t-tests for both equal and not equal (Welch correction) variance assumptions, the mean values for both TIE and TRD are significantly different. This is, in fact, supported by the significantly smaller P values than our 0.01 level and the t- value that confirms the significant difference between the three samples. In fact, from the results in Table 1 for the mean TIE and TRD differences for the lower and upper limits in the two-sample for the three confidence levels, we conclude that both the mean TIE and TRD for the 24-h treated group is significantly less than the 2-h treated group (which has a smaller value than the control group). Therefore, t-test statistics analyses further confirm that treatment has significantly increased the radio-sensitivity of the 4T1 cells.

**Table 2:** Hypothesis testing by two-sample t-test.

Control group vs 2-h treated group						
	TIE			TRD		
	t Stat.	DF	Prob > |t|	t Stat.	DF	Prob > |t|
Equal Variance	4.17	173.00	4.89E-05	5.09	173.00	9.43E-07
NOT Equal Variance	4.20	136.10	4.70E-05	5.11	165.04	8.88E-07
**Two-sample mean values difference**						
**Confidence level**	**Lower Limits**		**Upper Limits**	**Lower Limits**	**Upper Limits**	
90%	19.47		45.10	7.46	14.66	
95%	16.99		47.58	6.77	15.35	
95%	12.10		52.47	5.40	16.73	
**Control group vs 24-h treated group**						
Equal Variance	9.66	215.00	1.45E-18	15.80	215.00	7.70E-38
NOT Equal Variance	8.32	100.43	4.40E-13	13.95	111.00	3.63E-26
**Two-sample mean values difference**						
**Confidence level**	**Lower Limits**		**Upper Limits**	**Lower Limits**	**Upper Limits**	
90%	48.06		67.89	22.61	27.89	
95%	46.14		69.80	22.10	28.40	
95%	42.38		73.57	21.10	29.40	
**2-h treated group vs 24-h treated group**						
Equal Variance	6.90	212.00	5.88E-11	10.70	212.00	1.25E-21
NOT Equal Variance	6.27	124.76	5.40E-09	9.64	120.91	1.15E-16
**Two-sample mean values difference**						
**Confidence level**	**Lower Limits**		**Upper Limits**	**Lower Limits**	**Upper Limits**	
90%	19.54		31.84	11.10	16.38	
95%	18.35		33.03	11.57	16.80	
95%	16.01		35.37	10.74	17.64	

**Figure 4:**
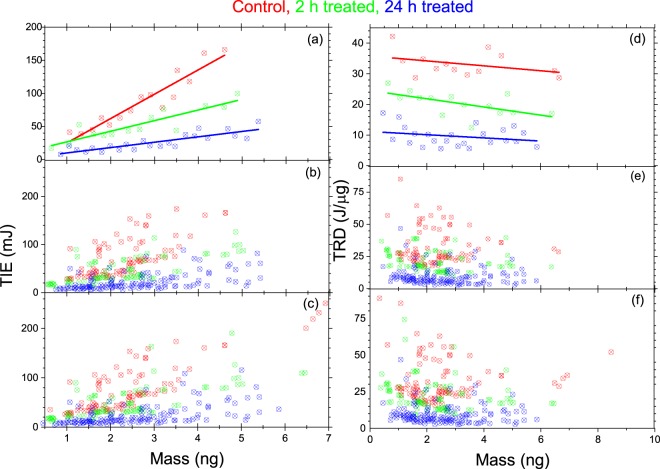
The TIE (**a–c**) and TRD (**d–f**) vs mass for untreated control (red), 2-h treated (green), and 24-h treated (blue) 4T1 breast cancer cells: (**c**) and (**f**) for all cells; (**b**) and (**e**) for the reduced data; and (**a**) and (**d**) is further reduced data with a linear fit.

In order to study the relationship between the ionization radiation energy and mass of the 4T1 cells, we have analyzed the TIE and TRD as a function of the mass of the individual cells. The results in Fig. 4(c–f) display the TIE and TRD versus mass for all 4T1 cells in the untreated control (red), 2-h treated (green), and 24-h treated (blue) groups. In order to establish a clear relationship between the TIE and TRD with the mass of the cells, we have made statistically valid data reduction using a graphical data analysis program software, Origin 2018. The reduced data is shown in Fig. 4(a,b) for the TIE and in Fig. 4(d,e) for the TRD. In this statistical reduction method, we first sort each data by the TIE or TRD in ascending order and eliminated the two minimum and the two maximum values from each of the three groups. We then made another sorting by mass in ascending order and again eliminated the two minimum and the two maximum values. The reduced data obtained following this procedure is shown in Fig. 4(b) for the TIE and in Fig. 4(e) for the TRD. Further reduction was made by subgrouping the data in Fig. 4(b–e) with 0.19 ng mass increment and calculating the average mass, TIE, and TRD for each subgroup. These results are shown in Fig. 4(a–d) for TIE and TRD, respectively. Figure 4(a) predicts an increase in TIE with an increase in the mass of the cells for all the three groups. It also consistently reconfirms the reduced TIE for the treated 4T1cells with the lowest for the 24-h treated group. On the other hand, although the TRD vs mass in Fig. 4(d) consistently confirms the low TRD for the treated group of cells, it predicts an inverse relationship between the TRD and the mass of the 4T1 cells in all three groups.

### Multiple cell ionization

In TIE and TRD studies for single cell, during the trapping process, we had to keep the cell isolated from the other cells until it is fully ionized and ejected from the trap. This was challenging as the ionization period is relatively longer for cancer cells compared to other small cells such as RBCs^18–20^. During this period, other nearby cells are attracted by the strong gradient force and jump into the trap at different times, which results in having multiple cells in the trap. Although these cells enter at different times into the trap, all the cells eject at the same time after ionization. In this section we wish to estimate the TIE and TRD when there are multiple cells in the trap for the untreated control, 2-h and 24-h treated groups. The purpose of this analysis is to study whether the TIE and TRD are affected by the addition of one or more cells while the cell that entered first undergoes membrane breakdown and charges continue to build up due to radiation damage. To accomplish this objective, in each of the untreated control groups, the 2-h and the 24-h treated groups, we formed five subgroups based on how many cells present in the trap during the ionization period: 1-cell, 2-cells, 3-cells, 4-cells, and 5-cells. The first group (1-cell) is what we already studied in the previous section and it is included here for comparison purposes. The images displayed in Fig. 2(II) show untreated 2-cells, 3-cells, 4-cells, and 5-cells ionization. Figure 2II(a) is the image captured when one cell is already in the trap (T) and the second is being accelerated towards the first. Figure 2II(b) is the image captured when both cells are inside the trap (T). Similarly, Fig. 2II(c) is when there are two cells in the trap and the third is moving towards; Fig. 2II(d) is when all three cells are in the trap (T); Fig. 2II(e) is when there are three cells in the trap and the fourth is on its way, and Fig. 2II(f) is when all four cells are in the trap (T). Next, Fig. 2II(g) is when there are three cells in the trap and two cells are on their way, and Fig. 2II(h) is when all five cells are in the trap (T). In multiple cell ionization, as we can see from Fig. 2, the cells do not always enter the trap at the same time, but all cells do eject from the trap at the same time. Therefore, the ionization period (T) is estimated for all subgroups using the images captured when the first cell enters the trap and the image when all cells eject from the trap. Then, the absorbed TIE is calculated using Eq. (2), following the same procedure we used for single cell ionization. In multiple cell ionization, the mass of the cells in the trap are also estimated using Eq. (1). However, because the mass of the cells in the trap is the sum of the masses of the individual cells in the trap with the same density, we replace *V*_*cell*_ by the *V*_*sum*_ which is the sum of the volume of the individual cells in the subgroups; hence, Eq. (1) becomes M_subgroup_ = ρ*V*_*sum*_. For example, for subgroup-2 (2-cells in the trap), we have *V*_*sum*_ = *V*_1_ + *V*_2_ and *M*_*subgroup*_ = ρ(*V*_1_ + *V*_2_) Then by replacing *M*_*cell*_ with *M*_*subgroup*_, we calculated the TRD for each subgroup in the untreated control, the 2-h, and 24-h treated groups.

**Figure 5:**
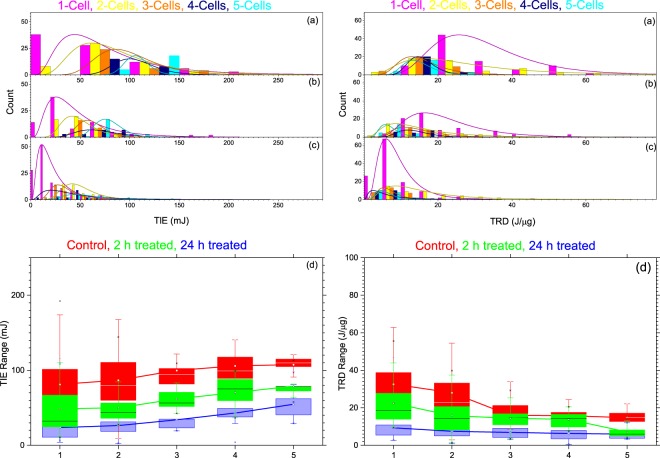
TIE (a-c, left column) and TRD (a-c, right column) in multiple cell ionization. In each column the statistical distribution displayed by the histograms (**a**) untreated control group, (**b**) 2-h treated, and (**c**) 24-h treated. In each of these histograms, the number of cells in the trap is represented by the color-coded legend: 1-cell (magenta), 2-cells (yellow), 3-cells (orange), 4-cells (navy), and 5-cells (Cyan). (**d**) displays the range of the TIE (left) and TRD (right) in each subgroup (1-cell, 2-cells, 3-cells, 4-cells, and 5-cells) for control (red), 2-h (green), and 24-h (blue) treated groups. The solid lines in (**d**) connect the average TIE (left) and TRD (right) for each subgroup in each group.

The results for the TIE and TRD for multiple cells along with single ionization are displayed using a histogram and the box-and-whisker plot in Fig. 5. The histograms on the left side of Fig. 5 are for the TIE, whereas the right side is for the TRD. In both left and right sides of Fig. 5(a) is for the untreated control group, Fig. 5(b) is for the 2-h treated group, and Fig. 5(c) is for the 24-h treated group. In each of these histograms, the subgroups 1-cell, 2-cells, 3-cells, 4-cells, and 5-cells are represented by pink, yellow, orange, navy, and cyan, respectively. From the distribution curves in the left side of Fig. 5(a–c), the shift in the peak values to the right indicates that the TIE increases as the number of cells increases in the trap consistently for control, 2-h, and 24-h treated groups. This is clearly shown in the box-and- whisker plot in Fig. 5(d). The summary for the values for the basic statistical parameters for the TIE is given in Table 3. The results clearly show that the average TIE for the five subgroups (1-cell to 5-cells) increases as the number of the cells increases consistently regardless of the treatment.

**Table 3:** The values for the basic descriptive statistical parameters for the TIE, the mass, and TRD for the five subgroups in the control untreated, 2-h treated, and 24-h treated groups of 4T1 cell-line.

Untreated 4T1 control group													
SG	N	TIE (mJ)				Mass (ng)				TRD (J/μg)			
		Mean	SD	Min.	Max.	Mean	SD	Min.	Max.	Mean	SD	Min	Max
1	89	81.3	63.5	24.9	440.1	2.5	1.4	0.3	8.5	32.6	14.8	15.8	88.6
2	54	86.8	37.7	9.28	200.9	3.5	1.1	1.3	5.4	28.1	17.2	2.9	82.5
3	34	99.7	34.8	59.1	193.6	5.1	1.4	2.7	8.0	16.0	6.5	8.4	34.0
4	23	106.0	21.4	75.7	140.4	6.9	1.0	5.1	9.1	15.8	3.9	11.3	26.0
5	23	107.4	11.1	80.1	121.1	7.7	1.9	5.1	11.0	14.7	3.8	8.3	22.0
**2-h treated 4T1 group**													
		**TIE (mJ)**				**Mass (ng)**				**TRD (J/μg)**			
1	86	48.8	37.7	10.5	192.2	2.3	1.3	0.4	6.5	22.4	11.4	9.4	55.6
2	49	50.1	20.7	27.8	144.2	4.2	3.4	1.3	20.6	15.8	9.1	1.5	39.8
3	30	61.9	14.9	42.4	109.3	4.9	2.4	2.9	14.7	14.5	5.6	3.6	29.3
4	20	70.4	19.9	36.5	98.9	5.5	2.7	3.7	11.7	13.8	4.1	7.5	20.5
5	23	78.9	14.7	57.6	113.1	8.2	2.7	1.0	12.0	7.1	3.1	5.3	78.7
**24-h treated 4T1 group**													
		**TIE (mJ)**				**Mass (ng)**				**TRD (J/μg)**			
1	128	23.8	20.4	4.0	115.5	2.6	1.2	0.5	5.9	9.3	6.4	2.7	46.9
2	56	26.2	11.9	2.7	61.9	3.9	1.5	1.1	9.0	7.5	4.4	1.0	25.3
3	30	34.0	14.4	19.3	63.7	5.1	1.6	3.3	9.4	6.9	2.8	3.2	11.9
4	21	43.1	18.3	4.3	81.1	7.7	2.6	4.1	14.7	6.3	3.8	0.6	16.3
5	20	54.7	19.9	29.0	97.1	9.5	2.0	5.4	8.1	6.0	2.6	3.3	12.0

It is important to make a quantitative comparison within the subgroups (1–5 cells) and between the three groups by analyzing the relative TIE percentage increase. For any of the three groups, putting aside any effects resulting from intracellular electrical and thermal interactions mediated by the radiation field, theoretically we expect 200–500% relative increase in the TIE for corresponding to 2–5 cells compared to the single cell. However, the calculated values were found to be 13.4%, 20.7%, 24.0%, and 29.3% (for untreated), 15.8%, 38.1%, 46.8% and 54.3% (for 2-h treated), and 18.8%, 35.3%, 54.1% and 59.8% (for 24-h treated). Although our estimation for the TIE in multiple cells ionization is by no means perfect, the results indicate an astounding intracellular electrical and thermal effect due to the infrared radiation used to trap the cells. We have also made a relative comparison between the treated groups with that of the control group for the corresponding multiple cells (2–5 cells) TIE. The calculated results were 42.3%, 37.9%, 35.6% and 26.5% for the 2-h treated and 47.7%, 45.1, 38.8% and 30.7% for the 24-h treated group, less than the corresponding values in the control group. These results reconfirm the increase in the radio sensitivity due to the treatment by DMDD that we discussed for single cells in the previous section.

The corresponding calculated TRD for multiple cells and for single cells is displayed using similar graphs and the same color coding on the right side of Fig. 5. Unlike the TIE, however, the distribution curves in all three groups–control (Fig. 5(a)), 2-h (Fig.5 (b)), and 24-h (Fig.5(c)) treated—shifts to the left, which indicates a decrease in TRD with an increase in the number of cells in the trap. This is clearly displayed in the box-and-whisker plot in Fig. 5(d) for control (red), 2-h (green), and 24-h treated (blue). The values for the basic statistical parameters in the TRD are also summarized in Table 3. The results show that as the number of cells in the trap increases from 1–5 cells, the average value for the TRD decreases consistently: 32.6–14.74 J/µg for the control: 22.4–7.1 J/µg for the 2-h treated; and 9.4–6.0 J/µg for the 24-h treated group. The relative decrease in multiple (2–5) cells’ average TRD as compared to single cells was found to be 13.9%, 51.1%, 51.7%, and 54.8% for untreated; 29.5%, 35.4%, 38.4%, and 66.7 for 2-h treated; and 20.1%, 25.9%, 32.8%, and 35.7% for 24-h treated.

**Figure 6:**
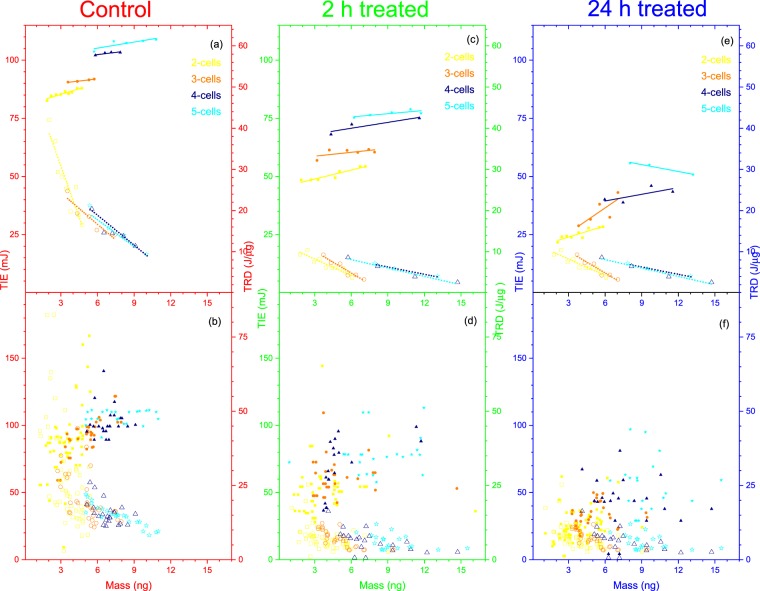
Multiple cell TIE and TRD vs mass for untreated control (left (**a,b**)), 2-h treated (middle (**c,d**)), and 24-h treated (right (**e,f**)). The number of cells in the trap is represented by the color coded legend: 2-cells (yellow), 3-cells (orange), 4-cells (navy), and 5-cells (Cyan). Each of these graphs are double-y axis graphs where the left axis represent the TIE and the right axis represent the TRD. The bottom graphs (**b**), (**d**), and (**f**) displays the complete data for each case of multiple cell ionization (2-cells (yellow), 3-cells (orange), 4-cells (navy), and 5-cells (Cyan)) for the TIE vs mass (Filled:: rectangle, circle, triangle, star) and the TRD vs mass (unfilled: rectangle, circle, triangle, star). The top row graphs display the corresponding reduced data with linear fit are for TIE (solid lines) and TRD (dotted line).

The results found for both the TIE and TRD multiple cells ionization (2–5 cells) in comparison with that of single cell predicts interesting physical processes that could have significant implications for radiation dosimetry, especially for combined modalities of cancer treatment that include chemo and possibly hyperthermia therapy. In order to explain these observed effects, following a similar statistically valid data reduction, we have examined how both TIE and TRD changes as a function of mass when multiple cells (2–5 cells) inter the trap. These results are shown using double-y axis graphs in Fig. 6 for the control ((a) and (b)), 2-h ((c) and (d)) and 24-h ((e) and (f) treated. In each of these graphs the left and the right axes represent the TIE and the TRD, respectively. Furthermore, in all these graphs the data points for TIE and the TRD for the number of cells in the trap are described by same color coding (2- cells (yellow), 3-cells (orange), 4-cells (Navy), and 5-cells (Cyan)) using different symbols (rectangle, circle, triangle, and star). These symbols are filled with the corresponding color for the TIE but not for the TRD. The graphs in the bottom row (b), (d), and (f) display all the calculated data for the TIE and TRD vs mass whereas the graphs in the top row (a), (c), and (e) display the reduced data with the linear fit that is obtained following a similar procedure we used for single cell. The linear fit (solid for TIE and dotted for TRD) to the reduced data in Fig. 6(a,c,e), consistent to the results for the single cell in Fig. 4, predicts an increase in the TIE and a decrease in the TRD with mass in multiple cell ionization in all three groups.

The low TRD we observed in multiple cell ionization could primarily be due to the gradual charging of the cell and elevated temperature resulting from the infrared ionization radiation. Radiation kills cancer cells by damaging their DNA. This can happen when the radiation incident on the cancer cells delivers enough amount of ionization energy resulting in the dielectric breakdown. It is well known that this minimum energy (TIE) or minimum radiation dose (TRD) can be affected when it is used along with chemo or hyperthermia. In the absence of the radiation, a simplistic physical model of a cell (in this case, a 4T1 cell) is a dielectric sphere filled with a collection of different electric dipoles. When it is exposed to a rapidly oscillating electric field like that of the laser radiation, these various dipoles respond differently by creating a stronger oscillation that aligns with the polarization direction of the radiation field. This leads to a stronger oscillating global polarization in the cell which, otherwise, is zero in the absence of the field. When the electric field becomes strong (high power radiation), the dipoles with weak strength start to break and free charges begin to develop in the cell. As the cell consists of different types of molecules characterized by different dipole strength, the different types of dipoles require different energy and break at different times. Consequently, the charge buildup is a gradual, time-dependent process. Furthermore, due to the Gaussian nature of the laser beam, different parts of the cell receive different electric field strengths (with the strongest at the center). This field gradient also contributes to the gradual charge buildup in the cell. This process could have a significant impact in the threshold radiation dose in multiple cell ionization when cells enter the trap at different times. When the first trapped cell undergoes dielectric breakdown by the radiation and the charge continues to build, any other cells entering the trap face the electric field of the charge on the first cell in addition to the rapidly oscillating electric field from the radiation. The electric field due to these free charges on the cell(s) that is (are) already in the trap causes extra damage in multiple cell ionization. Consequently, the TRD becomes smaller as the number of the cells increases. We have seen this consistently in all three groups. For example, in the untreated control group, the TRD which is measured by the ionization energy from the radiation (the laser): 5-cells (14.7 ± 3.8), <4-cells (15.8 ± 3.9), <3-cells (16.0 ± 6.5), <2-cells (28.1 ± 17.2), <1 cell (32.6 ± 14.8) in J/ μg.

The second major factor that could contribute to the lower TRD in multiple cell ionization is the hyperthermia effect. The radiation used to trap the cells is in the far infrared (1064 nm). The absorption of this wavelength by water molecules in the cell or in the surrounding suspension medium at such high power could be significant and it can significantly elevate the temperature in surrounding medium and the trapped cell. Therefore, when other cell(s) enter the trap, they face this elevated temperature. Studies have shown that at elevated temperature, 42–46 °C, cancer cells can die due to lack of glucose or the change in structure and enzymatic proteins^12^.

## Conclusion

We have studied the radio-sensitivity of 4T1 breast carcinoma cell lines treated by a naturally occurring antitumor compound, 2-Dodecyl-6-methoxycyclohexa-2, 5-diene-1, 4-dione (DMDD) extracted from the root of *Averrhoa carambola* L. The radio-sensitivity was studied by measuring and comparing the TIE and TRD for *in vivo* 2-h and 24-h treated groups with an untreated control group of 4T1 cells. The TIE and TRD were determined using a new approach that uses laser trapping technique for single and multiple cell ionization. The results obtained and confirmed by t-test statistical analyses show that TIE and TRD decreased as the period of treatment increased. This clearly demonstrates the increase in radio-sensitivity of the 4T1 cells due to the antitumor compound DMDD. The most interesting and significant part of the results is the TRD and its relation to the mass observed, in particular, in multiple cell ionization. These results demonstrate the significance of the effect of induced charges and hyperthermia resulting from radiation mediated electrical and thermal interactions within the cells. The results, regardless of the treatment, showed TRD decreased as the mass of the cell increased. This decrease in TRD becomes more significant in multiple cell ionization as we observed in TRD vs the number of cells entering the trap. In addition to the effect stemming from the antitumor compound used to treat the cells, the significant reduction in the TRD in multiple cell ionization is associated with the chain effect of ionization by the radiation field and the absorption by water molecules at 1064 nm^29^.

It is important to point out that, generally, the results reported in this study highlighted the effect of combined modalities in radiotherapy, chemo, and hyperthermia useful only *in vitro* cancer treatment. However, with the recent advances made in the development of biocompatible nanoparticles for combined modalities in cancer treatment, the method used and the results reported in this study could be applied for *in vivo* cancer treatment. Based on the results reported here, it is possible to develop a theoretical/computational model that predicts the TRD for *in vivo* treatment by extrapolating the TRD determined using multiple cell ionization if we have accurate information about the size and density of the tumor. However, this requires more advanced studies that integrate prior, during, and post ionization dynamics in both single and multiple cell ionization as well as the dose and period of the treatment by the antitumor compound, DMDD. One important prospective study is an accurate measurement of the charge and the temperature elevation that occurs when the cell(s) interact with radiation after a treatment.

## Data Availability

The datasets generated during and/or analyzed during the current study are available from the corresponding author on reasonable request.
